# Disruption of the hippocampal and hypothalamic blood–brain barrier in a diet-induced obese model of type II diabetes: prevention and treatment by the mitochondrial carbonic anhydrase inhibitor, topiramate

**DOI:** 10.1186/s12987-018-0121-6

**Published:** 2019-01-08

**Authors:** Therese S. Salameh, William G. Mortell, Aric F. Logsdon, D. Allan Butterfield, William A. Banks

**Affiliations:** 1 Geriatrics Research, Education and Clinical Center, Veterans Affairs Puget Sound Health Care System, 1660 S. Columbian Way, 810A/Bldg 1, Seattle, WA 98108 USA; 2 Division of Gerontology and Geriatric Medicine, Department of Medicine, University of Washington, Seattle, WA USA; 3 Department of Chemistry and Sanders-Brown Center on Aging, University of Kentucky, Lexington, KY USA

**Keywords:** Type II diabetes, Blood–brain barrier, Blood–retinal barrier, Hypothalamus, Hippocampus, Topiramate

## Abstract

**Background:**

Type II diabetes is a vascular risk factor for cognitive impairment and increased risk of dementia. Disruption of the blood–retinal barrier (BRB) and blood–brain barrier (BBB) are hallmarks of subsequent retinal edema and central nervous system dysfunction. However, the mechanisms by which diet or metabolic syndrome induces dysfunction are not understood. A proposed mechanism is an increase in reactive oxygen species (ROS) and oxidative stress. Inhibition of mitochondrial carbonic anhydrase (mCA) decreases ROS and oxidative stress. In this study, topiramate, a mCA inhibitor, was examined for its ability to protect the BRB and BBB in diet-induced obese type II diabetic mice.

**Methods:**

BBB and BRB permeability were assessed using ^14^C-sucrose and ^99m^Tc-albumin in CD-1 mice fed a low-fat (control) or a high-fat diet. Topiramate administration was compared to saline controls in both preventative and efficacy arms examining BRB and BBB disruption. Body weight and blood glucose were measured weekly and body composition was assessed using EchoMRI. Metabolic activity was measured using a comprehensive laboratory animal monitoring system. Brain tissues collected from the mice were assessed for changes in oxidative stress and tight junction proteins.

**Results:**

High-fat feeding caused increased entry of ^14^C-sucrose and ^99m^Tc-albumin into the brains of diet-induced obese type II diabetic mice. Increased permeability to ^14^C-sucrose was observed in the hypothalamus and hippocampus, and attenuated by topiramate treatment, while increased permeability to ^99m^Tc-albumin occurred in the whole brain and was also attenuated by topiramate. Treatment with topiramate decreased measures of oxidative stress and increased expression of the tight junction proteins ZO-1 and claudin-12. In the retina, we observed increased entry of ^99m^Tc-albumin simultaneously with increased entry into the whole brain during the preventative arm. This occurred prior to increased entry to the retina for ^14^C-sucrose which occurred during the efficacy arm. Treatment with topiramate had no effect on the retina.

**Conclusions:**

Blood–brain barrier and blood–retinal barrier dysfunction were examined in a mouse model of diet-induced obese type II diabetes. These studies demonstrate that there are spatial and temporal differences in ^14^C-sucrose and ^99m^Tc-albumin permeability in the brain and retina of diet-induced obese type II diabetic mice. Topiramate, a mitochondrial carbonic anhydrase inhibitor, is efficacious at both preventing and treating BBB disruption in this diet-induced obese type II diabetic mouse model.

## Introduction

Type II diabetes mellitus, as well as obesity and consumption of Western diets, are known vascular risk factors for cognitive impairment and increased risk of dementia [1, 2]. Emerging clinical evidence and studies in animal models of metabolic syndrome suggest that alterations in the integrity of the cerebrovascular blood–brain barrier (BBB) are associated with cognitive decline [3]. This phenomenon also occurs with visual impairment as observed with disturbances in blood–retinal barrier (BRB) integrity in diabetic retinopathy. The BBB and BRB provide a structural and functional barrier, which impedes and regulates the influx of compounds from the blood into the brain or retina, respectively. There is growing evidence suggesting that the disruption of the BRB and BBB is an early hallmark of the subsequent retinal edema and central nervous system (CNS) dysfunction [4, 5]. However, the mechanisms by which diet or metabolic syndrome induces dysfunction of the BBB and BRB are not completely understood.

High-fat (HF) diet consumption causes BBB disruption in a variety of animal models of obesity regardless of the diet compositions; examples include high energy diets and diets rich in saturated fatty acids [3, 6–8]. Type II diabetes, designated by hyperglycemia and insulin resistance, is associated with cognitive dysfunction characterized by microvascular and neurovascular unit changes. Changes in the brain microvasculature are characterized by altered permeability, cerebral extravasation of plasma molecules, neuroinflammatory and oxidative milieu, and progressive loss of neuronal function. These features are all associated with type II diabetes, although there are a limited number of studies examining the effects of diabetes on BBB integrity. In a clinical study, patients with type II diabetes showed increased BBB permeability to gadolinium diethylenetriamine pentaacetic acid (DTPA; 570 Da) by magnetic resonance imaging [9]. In an animal study, in high-fat diet fed insulin-resistant mice, BBB dysfunction was shown to precede cognitive decline and neurodegeneration [10]. In KKA^*y*^ mice, a model of type II diabetes, Min et al. demonstrated that blocking angiotensin II type 1 receptors to activate peroxisome proliferator-activated receptor (PPAR)-γ protected against cognitive decline by preserving the integrity of the BBB [11]. More recently, Rom et al. published a paper examining BBB disruption using the fluorescent tracer, sodium-fluorescein (NaF) in the leptin receptor deficient *db/db* mouse model [12]. These publications assessed BBB integrity using either non-quantitative techniques such as Evans Blue or IgG extravasation or non-reliable quantitative methods such as sodium fluorescein [13]. Here, we studied disruption of the BBB and BRB in a diet-induced obese model of type II diabetes using a quantitative approach of radiolabeled ligands of varying sizes.

Although there is a great deal of diversity in microvascular and macrovascular complications associated with type II diabetes, many arise through the same hyperglycemia-associated pathways mediated by the overproduction of superoxide [14, 15]. Increased oxidative stress is thought to precede the onset of high-fat diet induced insulin resistance and obesity [16]. In numerous studies, increased oxidative stress is a major driving force leading to BBB disruption and cognitive impairment [17–24]. Disruption to the brain microvasculature arises in streptozotocin (STZ)-induced rodent models of type I diabetes [25, 26]. Inhibition of mitochondrial carbonic anhydrase (mCA) has been shown to decrease reactive oxygen species (ROS) and oxidative stress in STZ-induced diabetic mouse brains [15, 27]. Topiramate (Topomax^®^; TPM), a mCA inhibitor, prevents BBB disruption in mouse models of type I diabetes [15, 25]. Also, TPM has been efficacious in improving cognitive function in diabetic animal models [28].

There are three critical characteristics of brain endothelium that establish the BBB: (i) tight junctions that restrict paracellular diffusion of molecules; (ii) a reduction in the number of endocytic vesicles and lower rates of transcytosis relative to peripheral vasculature, and (iii) saturable transport of molecules between blood and brain. Endothelial cells are connected by specific tight junction proteins, such as claudins, occludins and Zona occludens (ZO-1, ZO-2, and ZO-3) and exhibit specific transport mechanisms and pinocytic vesicles. High-fat diet consumption decreases expression of tight junction proteins leading to reduced BBB integrity in parts of the brain affected by the diet such as the hippocampus, but not the parietal cortex or in the whole brain [6, 29–31]. In addition, decreased tight junction expression has been observed in STZ-induced diabetic mice and the leptin-receptor deficient *db/db* mice [12, 32].

In this study, we examined the effects of HF-feeding on the BBB and BRB of outbred CD-1 mice. Changes in the BBB and BRB permeability were quantitatively assessed using two different size markers, ^99m^Tc-labeled albumin (65 kDa) and ^14^C-labeled sucrose (342 Da). We demonstrated that treatment with the mitochondrial carbonic anhydrase inhibitor (mCAi), TPM, was able to both prevent disruption from occurring and was efficacious as a treatment for BBB disruption in type II diabetes. We examined oxidative stress in this model by measuring markers of lipid peroxidation and protein oxidation and demonstrated that TPM was able to attenuate the oxidative stress induced in this model of type II diabetes mellitus. In addition, we examined changes in tight junction proteins and demonstrated that TPM treatment was able to prevent the loss of claudin-12 and ZO-1 after HF-diet consumption.

## Materials and methods

### Animals

Male CD-1 mice, 8 weeks of age, purchased from Charles River Laboratories (Wilmington, MA, USA), were used for all experiments. CD-1 mice have not been extensively utilized as a diet-induced obese model. However, as these are outbred mice it is important to study diet-induced obesity in them. In other mouse models, females have been found to be resistant to diet-induced obesity and less likely to develop hyperglycemia [33, 34], and therefore, for these studies we used male mice. Mice had ad libitum access to food and water and were placed on a 12-h light/dark cycle. All studies were performed under protocols in adherence with the Guide for the Care and Use of Laboratory Animals and approved by the Institutional Animal Care and Use Committee at the Veterans Affairs Puget Sound Health Care System (VAPSHCS). The data presented here is in compliance with the Animal Research: Reporting in Vivo Experiments (ARRIVE) guidelines.

### Study design

**Fig. 1:**
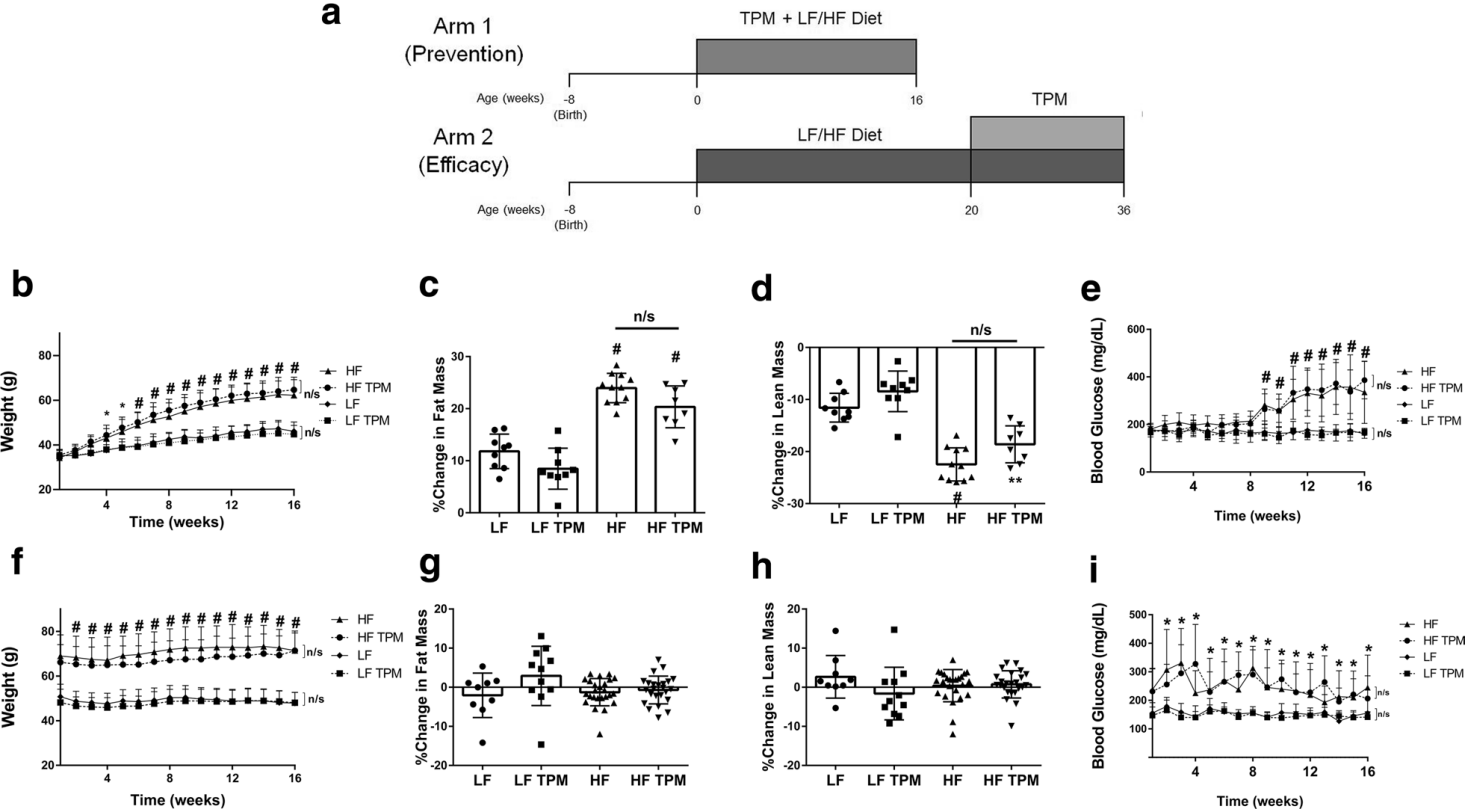
Effects of diet and topiramate treatment on body composition and blood glucose in CD-1 mice. A schematic diagram illustrating the difference in design between arm 1 and 2 of the study (**a**). Total body weight over the 16 weeks of TPM treatment for arm 1 (**b**; started with HF diet and thus prior to obesity) and arm 2 (**f**; started after establishment of obesity). HF consumption led to significant increases in body weight compared to LF diet in both arms. Topiramate treatment had no effect on weight gain. Change in fat mass as a percentage calculated using fat mass measured at the beginning and end of the treatment cycle for arm 1 (**c**) and arm 2 (**g**) of the study. Change in lean mass presented as a percentage change from the beginning of the treatment cycle for arm 1 (**d**) and arm 2 (**h**) of the study. Topiramate had no effect on lean and fat mass. Nonfasted measurements of blood glucose over the 16 week treatment period for arm 1 (**e**) and arm 2 (**i**). In both arms, HF feeding led to a significant increase in nonfasted blood glucose, which was not effected by TPM treatment. Values are expressed as mean ± SD. Significance was determined by a one-way analysis of variance followed by Newman–Keuls post-test for HF vs. LF, LF vs. LF TPM, and HF vs. HF TPM. *p < 0.05; **p < 0.01; ^#^p < 0.001

Male CD-1 mice (8 weeks of age; *n* = 235) were split into two arms, in the first arm, the prevention arm, mice were placed on their respective diets and TPM administered simultaneously (Fig. 1a). The mice were divided into 4 groups: low-fat (LF) diet + saline (*n* = 40), LF diet + TPM (*n* = 40), high-fat (HF) diet + saline (*n* = 40), HF-diet + TPM (*n* = 40). As this is an outbred model, we assumed that not all mice placed on HF diet would develop obesity and become hyperglycemic, thus a high *n* at the start was necessary to achieve statistical significance at the endpoint. Mouse diets were purchased from Research Diets, Inc. (High fat—D12492; Low fat—D12450J; New Brunswick, NJ, USA) and matched for sucrose (7%). For 16 weeks, topiramate (T0575; Sigma-Aldrich, St. Louis, MO, USA; TPM) was administered daily by subcutaneous injection at a dose of 5 mg/kg. This aligns with the dosing for therapeutic use in humans. Topiramate was dissolved in dimethylsulfoxide (DMSO) at 1:4 (wt/vol) and further dissolved in saline. Control mice were given an equivalent amount of DMSO in saline. Weekly weight measurement allowed for adjustment in dosing. Non-fasted blood glucose was measured weekly using the AlphaTRAK2 blood glucometer (Zoetis, Parsippany, New Jersey, USA) from the tail vein. High-fat fed mice were considered type II diabetic, and included in the analysis if their non-fasted blood glucose was 2.5 standard deviations above the mean for the LF-fed control mice. As a result, the permeability studies examining the brain and retina had an *n* in each group of: LF diet + saline (*n* = 10), LF diet + TPM (*n* = 10), HF diet + saline (*n* = 11), and HF diet + TPM (*n* = 8). Brain tissue and serum were collected for use in oxidative stress analysis, protein analysis, immunohistochemical analysis, and in the Bioplex Pro Mouse Diabetes assay. From this tissue collection, there was an *n* in each group of: LF diet + saline (*n* = 14), LF diet + TPM (*n* = 14), HF diet + saline (*n* = 17), and HF diet + TPM (*n* = 12). After adjustment for blood glucose, we had an *n* in each group of: LF diet + saline (*n* = 14), LF diet + TPM (*n* = 14), HF diet + saline (*n* = 7), and HF diet + TPM (*n* = 5).

In the second arm of the study, the efficacy arm (Fig. 1a), CD-1 mice were fed LF or HF diet for 20 weeks to develop obesity before they were started on TPM. There were 75 mice designated for use in this arm of the study. During the 20-week feeding period, mice were housed four to a cage, and body weight and non-fasted blood glucose were monitored monthly. After 20 weeks of being fed LF or HF diet, mice were split, two to a cage, and into four groups: LF diet + saline (*n* = 10), LF diet + TPM (*n* = 11), HF diet + saline (*n* = 27), and HF diet + TPM (*n* = 27). Mice were given an injection of saline ± TPM for an additional 16 weeks with weight and blood glucose measured weekly, while being maintained on their respective diets. After adjustment for blood glucose, mice used for permeability analysis totaled: LF diet + saline (*n* = 5), LF diet + TPM (*n* = 6), HF diet + saline (*n* = 10), and HF diet + TPM (*n* = 9). Brain tissue and serum were also collected from the second arm for use in oxidative stress, protein analysis, immunohistochemical analysis, and in the Bioplex Pro Mouse Diabetes assay. From this tissue collection, there was an n in each group of: LF diet + saline (*n* = 5), LF diet + TPM (*n* = 5), HF diet + saline (*n* = 17), and HF diet + TPM (*n* = 18). After adjustment for blood glucose, we had an *n* in each group of: LF diet + saline (*n* = 5), LF diet + TPM (*n* = 5), HF diet + saline (*n* = 15), and HF diet + TPM (*n* = 12).

Three mice died during feeding/treatment (LF diet + saline, *n* = 1, and HF diet + saline, *n* = 2) due to unrelated causes. Sixteen mice died during anesthesia (LF diet + saline, *n* = 1, LF diet + TPM, *n* = 4, HF diet + saline, *n* = 3, and HF diet + TPM, *n* = 8).

### Radiolabeled albumin and sucrose permeability experiments

Mice were anesthetized with an intraperitoneal (ip) injection of 40% urethane. Once anesthetized, mice received an injection into the jugular vein of 0.2 mL of 1% bovine serum albumin (BSA) lactated Ringer’s solution containing ^14^C-sucrose (10 × 10^6^ cpm/mouse) and ^99m^Tc-albumin (5 × 10^6^ cpm/mouse). To reduce exposure to ethanol (used by manufacturer to dissolve ^14^C-sucrose), the ethanol was evaporated in the fume hood and the ^14^C-sucrose resuspended in 1% BSA lactated Ringer’s solution. Recovery after evaporation results in 5–6 × 10^6^ cpm/mouse of ^14^C-sucrose being injected intravenously. Bovine albumin (A7030; Sigma-Aldrich, St. Louis, MO, USA) was radioactively labeled with ^99m^Tc using a stannous tartrate solution acidified with HCl (20 min room temperature incubation) then purified on a Sephadex G-10 column. Fractions were collected and assessed using 15% trichloroacetic acid. All proteins showed greater than 90% activity in the precipitate. The injection circulated for 30 min before the vascular space was washed out with ice-cold lactated Ringer’s solution (20 mL in 2 min). The brain was excised and dissected into: pons-medulla, midbrain, cerebellum, frontal cortex, occipital cortex, parietal cortex, thalamus, hypothalamus, hippocampus, and striatum. The retina and vitreous humor were also collected, as well as the olfactory bulbs. Serum was collected by centrifuging blood (3200 rpm; 10 min at 4 °C) isolated from the descending abdominal aorta.

^99m^Tc-albumin radioactivity was measured using a gamma counter (Wizard2; PerkinElmer, Shelton, CT, USA; 3 min counts). The samples were then processed for measuring in the beta counter (TriCarb 3110TR; PerkinElmer; 60 min counts). Data is expressed as a ratio of the amount of radioactivity measured in the tissue and serum (T/S) in units of µL/g calculated by the following equation:

$${\text{T}}/{\text{S }(g/\mu\text L)} = \left( {{\text{cpm}}/{\text{g brain tissue}}} \right) / \left( {{\text{cpm}}/\upmu{\text{L serum}}} \right)$$

#### Body composition

A EchoMRI 4-in-1 instrument (Echo Medical Systems, Houston, TX, USA) used quantitative magnetic resonance (QMR) to determine lean body and fat mass of the mice at the beginning and end of the study. Triplicate measurements were taken using plastic restrainer tubes with unanaesthetized mice. The procedure was performed by the Rodent Metabolic and Behavioral Phenotyping Core at the VAPSHCS.

#### Metabolic activity

Metabolic parameters, physical activity, and food intake were measured in male CD-1 mice (n = 8 per group) as previously described [35]. Animals were monitored for 72 h (12 h light/dark cycle at thermoneutrality 30 °C) in a comprehensive laboratory animal monitoring system (CLAMS; Columbus Instruments, Columbus OH, USA). Data was collected in 20 min intervals to measure oxygen consumption (VO_2_), respiratory quotient (RQ), energy expenditure, food intake, total physical activity, and ambulatory activity. Mice were administered saline or 5 mg/kg TPM at 10 a.m. daily.

#### Bio-plex pro mouse diabetes assay

A collection of metabolism-related factors [ghrelin, glucose-dependent insulinotropic peptide (GIP), glucagon-like peptide-1 (GLP-1), glucagon, insulin, leptin, plasminogen activator inhibitor-1 (PAI-1), and resistin] were measured in serum samples collected from LF- and HF-fed mice treated with saline or 5 mg/kg TPM using the murine Bioplex Pro diabetes assay kit according to the manufacturers protocol (Bio-Rad Laboratories, Hercules, CA, USA). We pooled data from animals in arm 1 with arm 2 as there was no significant difference between the groups. For example, LF arm 1 was pooled with LF arm 2 after t-test was completed to show they were not statistically different. After adjusting for blood glucose levels, we obtained the following n in each group: LF diet + saline (n = 10), LF diet + TPM (n = 11), HF diet + saline (n = 8), and HF diet + TPM (n = 8).

#### Oxidative stress measurements

To examine oxidative stress in this model, we measured levels of protein carbonyls, 3-nitrotyrosine (3-NT) and 4-hydroxynonenal (HNE) as previously described [36]. Protein carbonyls and 3-NT are measurements of protein oxidation and HNE is a measurement of lipid oxidation. These measurements were determined immunochemically. Protein samples (250 ng) from the brains of CD-1 mice fed LF- and HF-diet with and without TPM treatment were blotted onto a nitrocellulose membrane with a slot blot apparatus. In the case of protein carbonyls, protein samples were derivatized by 2,4-dinitrophenylhydrazine (DNPH) to react with protein carbonyls to form protein hydrazones as previously described [29]. The membranes were incubated with rabbit polyclonal anti-DNPH antibody (1:100 dilution) to measure protein carbonyls, anti-nitrotyrosine antibody (1:200 dilution) and anti-4-hydroxynonenal polyclonal antibody (1:5000 dilution). Blots were developed using fast tablet (BCIP/NBT; Sigma-Aldrich) and quantified using Scion Image (PC version of Macintosh- compatible NIH image) software. No non-specific background binding of the primary or secondary antibodies was found.

### Confocal microscopy of tight junction proteins

Dissected mouse brains were immediately post-fixed in 4% paraformaldehyde in PBS at 4 °C and then equilibrated with cryoprotectant (30% sucrose in PBS) and stored at 4 °C for up to 6 months until analyzed. Sagittal sections embedded in OCT (Tissue-Tek, Torrance, CA, USA) were cut at 50 μm thickness from bisected brains with a CM1850UV cryostat (Leica, Buffalo Grove, IL, USA). Antigen retrieval was performed by washing the sections in PBS (3 × 5 min), transferring them to 50 mM sodium citrate (pH 9.0) and then heating at 80 °C for 30 min. Sections cooled to room temperature were washed (3 × 5 min) in PBS. A mouse on mouse kit (Vector Laboratories, Burlingame, CA, USA) was used in accordance with the manufacturer’s recommendations. The following antibodies were applied overnight at 4 °C: mouse monoclonal anti-occludin (Invitrogen, Carlsbad, CA; 1:100), rabbit monoclonal anti-caveolin-1 (D46G3; Cell Signaling, Danvers, MA; 1:1000), rat anti-ZO-1 (Clone R40.76; Millipore Sigma, Burlington, MA; 1:100), rabbit anti-GLUT-1 (Millipore Sigma; 1:1000). Goat secondary antibodies labeled with Alexa Cy3 or Alexa Fluor^®^ 488 were applied for 2 h (Jackson Immunoresearch, West Grove, PA, USA; 1:1000). Floating tissue sections were cover slipped with a drop of Prolong^®^ Gold Antifade Reagent with or without DAPI (Invitrogen, Eugene, OR, USA). Confocal microscopy was performed on the hypothalamus with a Leica TCS SP5 II microscope with Leica objectives (20× and 0.7 numerical aperture). All confocal microscopic images were acquired and processed using image adjustments limited only to linear contrast and brightness adjustments applied identically between and within groups.

#### Immunoblot analysis

Whole cell lysate extracts were prepared by lysing hemibrains in lysis buffer (25 mM Tris, pH 7.5, 0.15 M NaCl, 1 mM phenylmethylsulfonylfluoride, 1% Triton X-100 and complete protease and phosphatase inhibitor cocktails, Sigma-Aldrich, Inc.; St. Louis, MO, USA) using a beadbeater (2 pulses for 30 s each at 4500 rpm). Cell debris was removed by centrifugation at 14,000 rpm for 20 min at 4 °C. Amount of protein in each sample was determined using the BCA protein assay kit (Pierce; Rockford, IL, USA). 20 µg of protein lysate was subjected to electrophoresis in denaturing 4–12% SDS-PAGE. Membranes were probed for caveolin-1 (1:1000; Cell Signaling Technology; Beverly, MA, USA), claudin-5 (1:1000; ThermoFisher Scientific; Waltham, MA, USA), claudin-12 (1:1000; ThermoFisher Scientific; Waltham, MA, USA) and ZO-1 (1:1000; Invitrogen; Carlsbad, CA, USA) overnight at 4 °C. As a loading control, membranes were probed for β-actin (1:10,000). The enhanced chemiluminescence western blot was digitalized with a LAS4000 CCD imaging system (GE Healthcare, USA) and analyzed by ImageQuant TL software.

#### Statistical analysis

Results are expressed as a mean ± standard deviation. Statistical analysis was done on the groups using analysis of variance (ANOVA) followed by Newman–Keuls post-test.

## Results

### Topiramate has no effect on body composition and non-fasted blood glucose levels

Male CD-1 mice (Charles River Laboratories; 8 weeks of age) had a baseline weight of 34.21 ± 0.17 g. Mice were placed in one of two arms: arm one, examining the role of TPM in prevention of BBB dysfunction, started 16-week TPM treatment or vehicle simultaneously with the change in diet; arm two, examining the efficacy of TPM as treatment in reversing BBB disruption, started TPM treatment after 20 weeks of diet consumption (when BBB disruption is known to be present), continuing mice on the assigned diet for an additional 16 weeks.

After 4 weeks of consumption, mice fed a HF-diet showed a significant increase in body weight compared to mice on a LF-diet (Fig. 1b). This was maintained until the study endpoint. Consistent with the literature, TPM treatment had no effect on body weight and body composition in these mice (Fig. 1b–d). The basal non-fasted blood glucose of the CD-1 mice was 178.68 ± 2.24 mg/dL. After 9 weeks on HF-diet, CD-1 mice showed a significant increase in non-fasted blood glucose compared to LF-diet fed mice (167.2 ± 10.85 mg/dL in LF vs. 281.9 ± 20.29 mg/dL in HF; p < 0.0001) (Fig. 1e). There was no significant difference in non-fasted blood glucose between saline and TPM treated mice on either the LF- or HF-diet. CD-1 mice on LF-diet had a baseline weight of 49.33 ± 1.31 g, while those on HF-diet weighed 67.39 ± 1.19 g (p < 0.0001) after 20 weeks on their respective diets. There was no significant difference in body weight between saline and TPM treated mice on either diet throughout the second arm (Fig. 1f). There was no effect on fat mass (Fig. 1g) or lean mass (Fig. 1h) in these animals. In this arm of the study, LF-fed mice had a basal non-fasting blood glucose of 150.9 ± 2.6 mg/dL, while those on the HF-diet had a significantly higher basal non-fasting blood glucose of 250.05 ± 9.35 mg/dL (p < 0.05). Topiramate treatment had no effect on blood glucose levels (Fig. 1i).

### Topiramate has no effect on metabolic activity and metabolism-related hormones

**Fig. 2:**
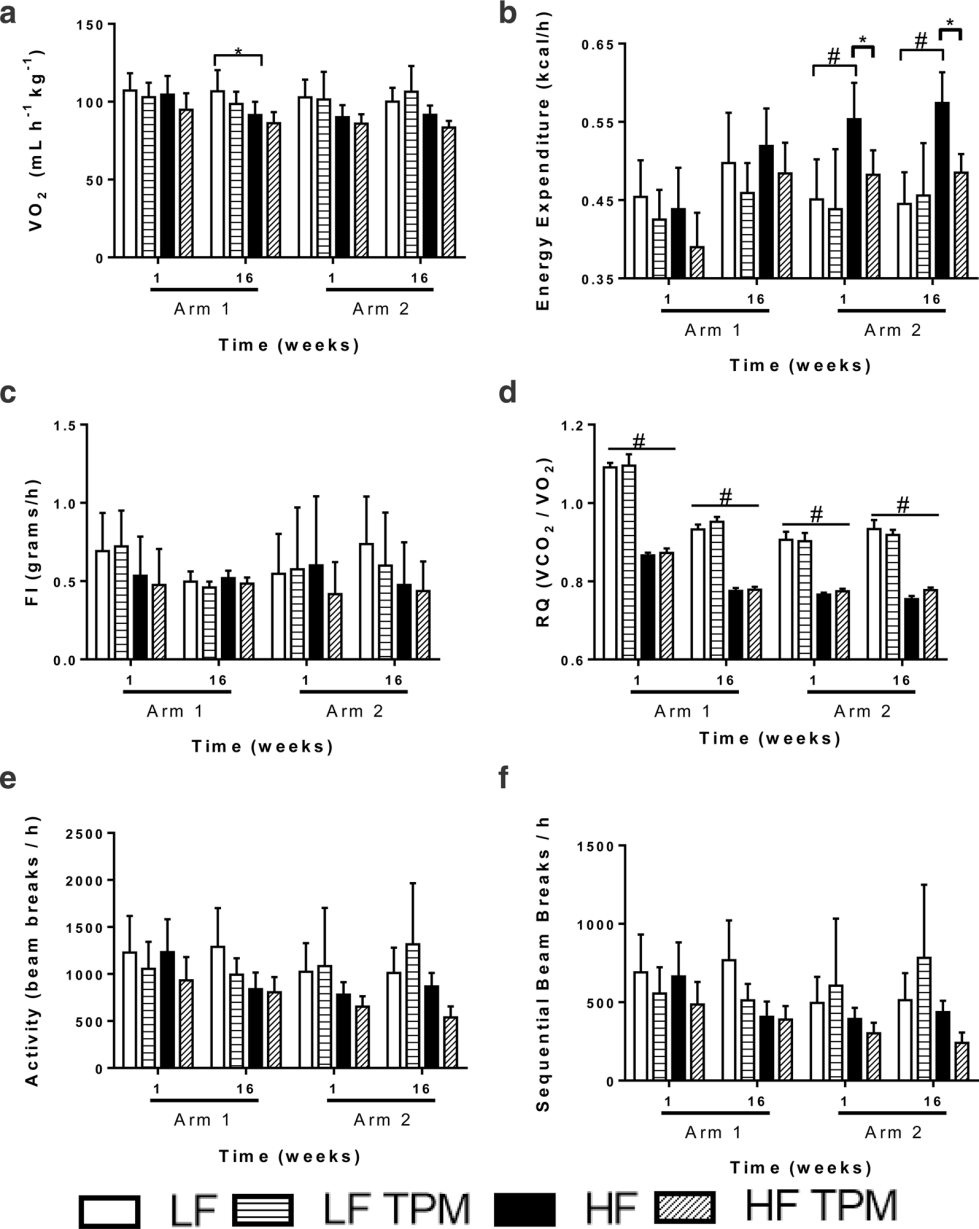
Metabolic activity of topiramate-treated CD-1 mice. Metabolic data collected from the laboratory animal monitoring system (CLAMS) after 1 week and 16 weeks of TPM administration as a preventative (arm 1) or efficacy (arm 2) agent from LF diet + saline (n = 8), LF diet + TPM (n = 8), HF diet + saline (n = 8), and HF diet + TPM (n = 8) in each arm. Mice were administered treatments once daily and measured for 72 h. Data collected includes **a** oxygen consumption (VO_2_; mL/h/kg), **b** energy expenditure (kcal/h), **c** food intake (FI; grams/h), **d** respiratory quotient (RQ: VCO_2_/VO_2_), **e** total activity (beam breaks/h), and **f** ambulatory activity (sequential beam breaks/h). Values are expressed as mean ± SD. Significance was determined by a one-way analysis of variance followed by Newman–Keuls post-test between HF vs. LF, LF vs. LF TPM, and HF vs. HF TPM. *p < 0.05; ^#^p < 0.001

**Fig. 3:**
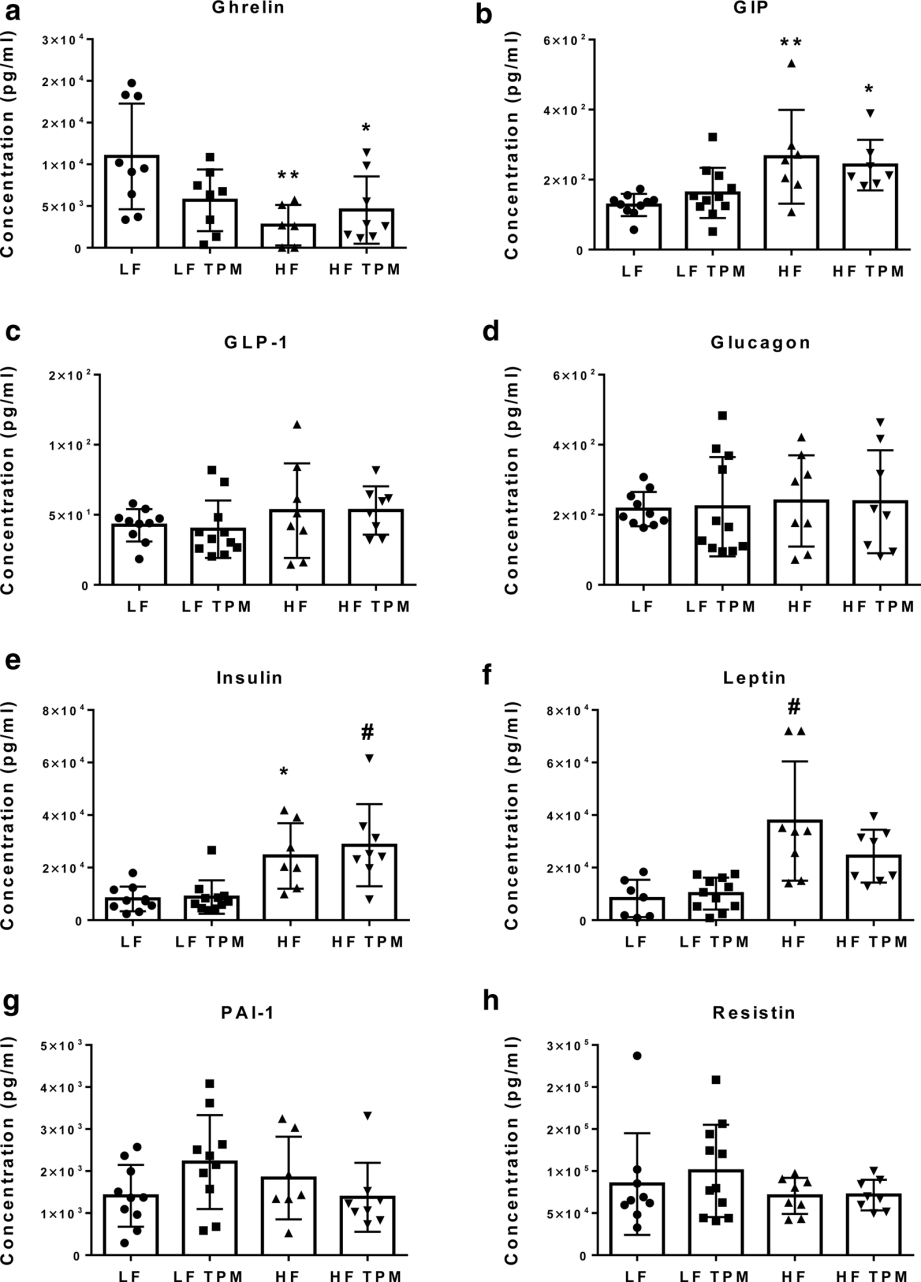
Effect of diet and topiramate on metabolism-related hormones. Serum samples collected from LF diet + saline (n = 10), LF diet + TPM (n = 11), HF diet + saline (n = 8), and HF diet + TPM (n = 8). Serum was measured for **a** ghrelin, **b** glucose-dependent insulinotropic peptide, GIP, **c** glucagon-like peptide-1, GLP-1, **d** glucagon, **e** insulin, **f** leptin, **g** plasminogen activator inhibitor-1, PAI-1, and **h** resistin. Ghrelin showed a significant decrease after HF-feeding, while GIP, insulin, and leptin showed a significant increase. Topiramate treatment had no significant effect at either low- or high-fat feeding. Values are expressed as mean ± SD. Significance was determined by a one-way analysis of variance followed by Newman–Keuls post-test between HF vs. LF, LF vs. LF TPM, and HF vs. HF TPM. *p < 0.05; **p < 0.01; ^#^p < 0.001

Low- and high-fat fed CD-1 mice were placed into metabolic chambers after treatment with TPM for 1 week or 16 weeks in both arm 1 and arm 2 (Fig. 2). Topiramate treatment had no effect on oxygen use (Fig. 2a), food intake (Fig. 2c), or activity (Fig. 2e, f). An RQ of 0.7 indicates preferential utilization of lipids, whereas a value of 1.0 indicates use of carbohydrates. Topiramate treatment did not affect the RQ of animals on the respective diets (Fig. 2d). In arm 2, HF-diet consumption resulted in a significant increase (p < 0.0001) in energy expenditure compared to LF-fed controls (Fig. 2b). This increase was significantly attenuated with TPM treatment. Analysis of serum metabolites showed that HF-feeding led to a significant decrease in ghrelin levels (Fig. 3a) and a significant increase in GIP (Fig. 3b), insulin (Fig. 3e) and leptin (Fig. 3f). HF-feeding caused no significant change in the level of GLP-1, glucagon, PAI-1, and resistin compared to the LF-treated group. Treatment with TPM had no significant effect on any of the metabolites measured compared to their LF and HF-treated counterparts (Fig. 3).

### High-fat diet consumption results in BBB permeability to ^99m^Tc-albumin which is attenuated by topiramate treatment

**Fig. 4:**
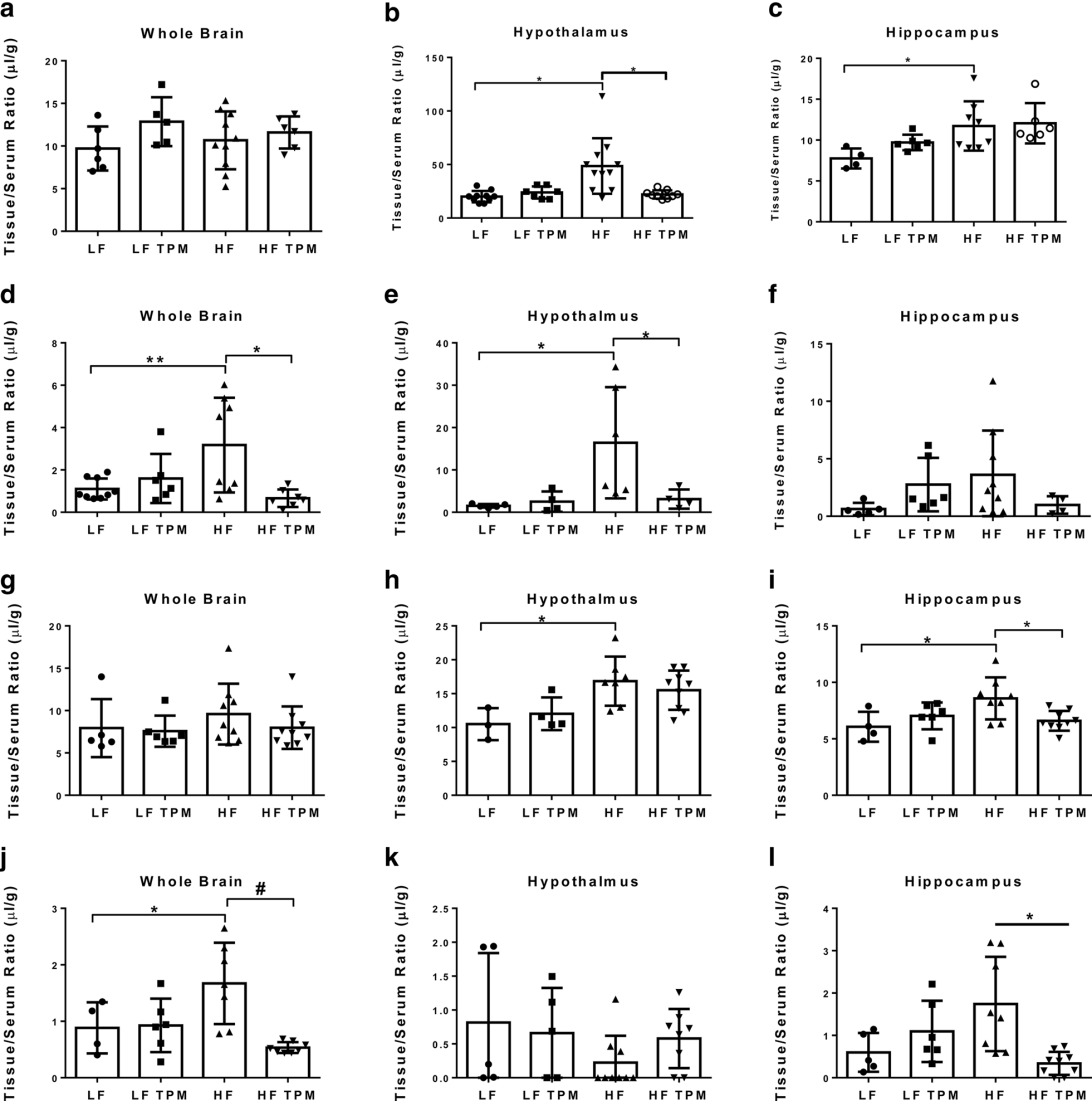
Measurement of blood–brain barrier permeability changes in diet-induced obesity. These histograms are representative of data highlighted in Tables 1 and 2. ^14^C-labeled sucrose (342 Da; Table 2) was used to measure BBB permeability changes in the whole brain (**a** prevention; **g** efficacy), hypothalamus (**b** prevention; **h** efficacy), and hippocampus (**c** prevention; **i** efficacy). ^99m^Tc-labeled albumin (65 kDa; Table 1) was used to measure BBB permeability changes in the whole brain (**d** prevention; **j** efficacy), hypothalamus (**e** prevention; **k** efficacy), and hippocampus (**f** prevention; **l** efficacy). Values are expressed as mean ± SD. Significance was determined by a one-way analysis of variance followed by Newman–Keuls post-test for HF vs. LF, LF vs. LF TPM, and HF vs. HF TPM. *p < 0.05; **p < 0.01; ^#^p < 0.001

Blood–brain barrier dysfunction was measured using ^99m^Tc-albumin (65 kDa). Results indicate that at 16 weeks in arm 1, the whole brain shows increased permeability to ^99m^Tc-albumin in the HF-fed animals (1.102 ± 0.495 µL/g in LF vs. 3.174 ± 2.237 µL/g in HF; p < 0.01) (Fig. 4d; Table 1). This observation was made at the whole brain level, and specifically in the hypothalamus (Fig. 4e; p < 0.05) and cerebellum (p < 0.05), with other regions trending towards significance (frontal cortex, parietal cortex, occipital cortex, and pons-medulla; p < 0.10). Topiramate treatment was able to attenuate BBB disruption in the whole brain (3.17 ± 0.79 µL/g in HF vs. 0.667 ± 0.413 µL/g in HF TPM; p < 0.01) (Fig. 4e; Table 1). Similar findings were observed in the whole brain after 36 weeks of HF-feeding (0.883 ± 0.452 µL/g in LF vs. 1.672 ± 0.719 µL/g in HF; p < 0.05, Fig. 4j; Table 1). At this time point, the hippocampus showed significant increased permeability (Fig. 4f; p < 0.05), with other regions trending towards significance (frontal cortex, parietal cortex, occipital cortex, thalamus, and midbrain; p < 0.10). Topiramate treatment also attenuated BBB permeability at 36 weeks in arm 2 (1.672 ± 0.719 µL/g in HF vs. 0.534 ± 0.096 µL/g in HF TPM; p < 0.001, Fig. 4j; Table 1). HF-feeding also led to increased permeability to ^99m^Tc-albumin in the retina at 16 weeks (18.20 ± 7.654 µL/g in LF vs. 38.46 ± 20.53 µL/g in HF; p < 0.05) and 36 weeks (19.27 ± 7.943 µL/g in LF vs. 36.40 ± 11.65 µL/g in HF; p < 0.05, Table 1). Topiramate did not attenuate the effects of HF-feeding in the retina and vitreous humor.

**Table 1:** Blood–brain barrier and blood–retinal barrier disruption to ^99m^Tc-albumin

Region	Prevention				Efficacy			
	LF	LF TPM	HF	HF TPM	LF	LF TPM	HF	HF TPM
Whole brain	1.102 ± 0.495	1.599 ± 1.160	3.174 ± 2.237^a^	0.667 ± 0.413^b^	0.883 ± 0.452	0.927 ± 0.473	1.672 ± 0.719^a^	0.534 ± 0.096^b^
Retina	18.20 ± 7.654	21.37 ± 8.186	38.46 ± 20.53^a^	29.98 ± 21.77^a^	19.27 ± 7.943	31.13 ± 8.985	36.40 ± 11.65^a^	29.62 ± 8.061
Vitreous humor	3.941 ± 1.141	3.591 ± 1.513	6.062 ± 1.613^a^	6.277 ± 2.636^a^	5.398 ± 2.516	8.791 ± 4.617	7.917 ± 5.659	6.694 ± 3.447
Olfactory bulb	2.110 ± 1.677	1.701 ± 0.789	2.267 ± 1.871	2.696 ± 1.483	1.697 ± 0.392	1.326 ± 0.653	2.383 ± 1.321	1.486 ± 0.547
Striatum	1.892 ± 0.995	1.746 ± 1.905	1.840 ± 1.167	1.976 ± 1.669	0.795 ± 0.770	0.681 ± 0.512	0.823 ± 0.776	0.412 ± 0.273
Frontal cortex	0.861 ± 0.815	1.081 ± 0.799	2.079 ± 1.736	0.949 ± 0.589	0.752 ± 0.344	0.773 ± 0.356	1.241 ± 0.765	0.522 ± 0.090^b^
Hypothalamus	1.528 ± 0.411	2.506 ± 2.400	16.41 ± 13.15^a^	3.123 ± 2.274^b^	0.815 ± 1.025	0.659 ± 0.667	0.223 ± 0.397	0.579 ± 0.436
Hippocampus	0.630 ± 0.538	2.759 ± 2.324	3.603 ± 3.855	0.984 ± 0.765	0.598 ± 0.458	1.094 ± 0.723	1.742 ± 1.112^a^	0.339 ± 0.273^b^
Thalamus	1.196 ± 0.640	1.951 ± 1.424	3.094 ± 3.046	1.273 ± 1.366	0.594 ± 0.510	0.710 ± 0.316	1.328 ± 1.258	0.367 ± 0.219
Parietal cortex	1.119 ± 0.665	1.615 ± 1.486	2.576 ± 2.362	0.871 ± 0.802	0.723 ± 0.412	0.745 ± 0.411	1.359 ± 1.050	0.371 ± 0.125^b^
Occipital cortex	1.314 ± 0.841	1.982 ± 1.370	2.817 ± 2.521	0.655 ± 0.406	1.070 ± 0.530	0.669 ± 0.547	1.103 ± 0.720	0.406 ± 0.233^b^
Cerebellum	0.738 ± 0.430	1.087 ± 1.029	2.851 ± 2.483^a^	0.990 ± 1.150	1.072 ± 0.742	0.899 ± 0.470	0.908 ± 0.458	0.752 ± 0.495
Midbrain	0.947 ± 0.825	1.908 ± 0.943	2.994 ± 2.301	1.936 ± 2.206	0.515 ± 0.288	0.799 ± 0.482	1.260 ± 0.791	0.289 ± 0.220^b^
Pons-medulla	1.472 ± 0.654	2.191 ± 1.267	3.057 ± 1.969	2.548 ± 1.490	1.361 ± 0.670	1.176 ± 0.420	1.671 ± 0.800	1.598 ± 2.272

^99m^Tc-albumin was used to assess permeability after TPM treatment

Units, µL/g. Values are expressed as mean ± SD. Significance was determined by a one-way analysis of variance followed by Newman–Keuls post-test

*LF* low-fat diet + saline group, *LF TPM* low-fat diet + topiramate group, *HF* high-fat diet + saline group, *HF TPM* high-fat diet + topiramate group

^a^Significance in comparison to LF control

^b^Significance in comparison to HF

### High-fat diet consumption results in hypothalamic and hippocampal increased permeability to ^14^C-sucrose which is attenuated by topiramate treatment

Blood–brain barrier permeability was measured using ^14^C-sucrose (342 Da). In Table 2, we show data collected to examine the ability of TPM to prevent BBB disruption in the brain. Results indicate that at 16 weeks of arm 1 (prevention), the brain shows increased permeability to ^14^C-sucrose in the hypothalamus (21.42 ± 6.130 µL/g in LF vs. 50.96 ± 27.71 µL/g in HF; p < 0.05; Fig. 4b) and hippocampus (7.753 ± 1.220 µL/g in LF vs. 11.73 ± 3.021 µL/g in HF; p < 0.05; Fig. 4c) (Table 2). Topiramate treatment was able to attenuate the increased permeability to ^14^C-sucrose in the hypothalamus (50.96 ± 27.71 µL/g in HF vs. 23.12 ± 4.293 µL/g in HF TPM; p < 0.05, Table 2). Also shown in Table 2 is data collected to determine the efficacy (arm 2) of TPM as a treatment for BBB disruption. Similar to our findings in prevention (arm 1), at 36 weeks the brain showed increased permeability to ^14^C-sucrose in the hypothalamus (10.51 ± 2.365 µL/g in LF vs. 16.85 ± 3.627 µL/g in HF; p < 0.05; Fig. 4h) and hippocampus (6.070 ± 1.327 µL/g in LF vs. 8.580 ± 1.862 µL/g in HF; p < 0.05; Fig. 4i, Table 2). Topiramate treatment was able to attenuate BBB disruption to ^14^C-sucrose in the hippocampus (8.580 ± 1.862 µL/g in HF vs. 6.594 ± 0.875 µL/g in HF TPM; p < 0.05, Table 2). At 36 weeks, the retina (138.1 ± 27.51 µL/g in LF vs. 184.1 ± 25.68 µL/g in HF; p < 0.05, Table 2) also showed increased permeability to ^14^C-sucrose which was not attenuated with TPM treatment.

**Table 2:** Blood–brain barrier and blood–retinal barrier disruption to ^14^C-sucrose

Region	Prevention				Efficacy			
	LF	LF TPM	HF	HF TPM	LF	LF TPM	HF	HF TPM
Whole brain	9.704 ± 2.586	12.86 ± 2.869	10.67 ± 3.386	11.59 ± 1.884	7.936 ± 3.422	7.568 ± 1.839	9.584 ± 3.593	7.978 ± 2.508
Retina	161.2 ± 38.52	153.4 ± 52.74	161.3 ± 30.87	197.2 ± 42.03	138.1 ± 27.51	166.2 ± 20.10	184.1 ± 25.68^a^	184.1 ± 31.34^a^
Vitreous humor	55.53 ± 18.10	51.12 ± 15.29	48.40 ± 13.74	65.70 ± 16.15	40.69 ± 1.939	59.70 ± 17.77	55.29 ± 14.97	60.10 ± 15.10
Olfactory bulb	18.58 ± 6.127	26.77 ± 14.24	23.47 ± 11.87	25.26 ± 13.44	15.74 ± 2.848	17.04 ± 4.781	17.38 ± 4.052	16.57 ± 2.744
Striatum	9.636 ± 2.148	9.643 ± 1.677	11.16 ± 3.700	11.58 ± 2.340	7.375 ± 2.047	6.979 ± 1.390	6.956 ± 1.072	7.978 ± 2.517
Frontal cortex	9.605 ± 3.311	11.22 ± 3.896	9.428 ± 4.862	11.09 ± 2.339	5.973 ± 1.285	6.161 ± 0.789	6.422 ± 1.225	6.029 ± 0.578
Hypothalamus	21.42 ± 6.130	24.46 ± 6.736	50.96 ± 27.71^a^	23.12 ± 4.293^b^	10.50 ± 2.365	12.04 ± 2.413	16.85 ± 3.627^a^	15.51 ± 2.898^a^
Hippocampus	7.753 ± 1.220	9.704 ± 0.950	11.73 ± 3.021^a^	12.06 ± 2.466^a^	6.070 ± 1.327	7.029 ± 1.186	8.580 ± 1.862^a^	6.594 ± 0.875^b^
Thalamus	10.05 ± 2.202	10.41 ± 2.625	8.770 ± 3.176	10.54 ± 2.138	7.135 ± 0.815	7.107 ± 1.435	6.421 ± 0.632	6.402 ± 0.674
Parietal cortex	8.412 ± 2.212	10.78 ± 3.310	9.458 ± 3.105	10.79 ± 2.743	6.235 ± 1.416	6.235 ± 1.369	6.413 ± 1.840	5.763 ± 0.827
Occipital cortex	9.764 ± 2.013	12.10 ± 3.037	10.31 ± 2.756	10.53 ± 2.759	7.815 ± 1.858	6.898 ± 1.683	7.451 ± 1.669	7.581 ± 1.401
Cerebellum	8.223 ± 2.127	10.12 ± 2.517	9.427 ± 3.065	10.20 ± 1.709	6.832 ± 1.551	6.890 ± 1.142	8.018 ± 3.389	6.654 ± 1.005
Midbrain	8.889 ± 3.054	13.09 ± 5.116	10.51 ± 3.445	12.87 ± 2.407	7.309 ± 1.458	7.321 ± 1.706	7.913 ± 1.991	6.774 ± 1.046
Pons-medulla	11.95 ± 4.057	13.90 ± 3.024	11.78 ± 4.316	14.71 ± 3.700	9.642 ± 1.321	10.92 ± 2.397	11.92 ± 6.198	10.24 ± 2.984

^14^C-sucrose was used to assess permeability after TPM treatment

Units, µL/g. Values are expressed as mean ± SD. Significance was determined by a one-way analysis of variance followed by Newman–Keuls post-test

*LF* low-fat diet + saline group, *LF TPM* low-fat diet + topiramate group, *HF* high-fat diet + saline group, *HF TPM* high-fat diet + topiramate group

^a^Significance in comparison to LF control

^b^Significance in comparison to HF

### Topiramate treatment decreases oxidative stress levels in high-fat diet fed mice

**Fig. 5:**
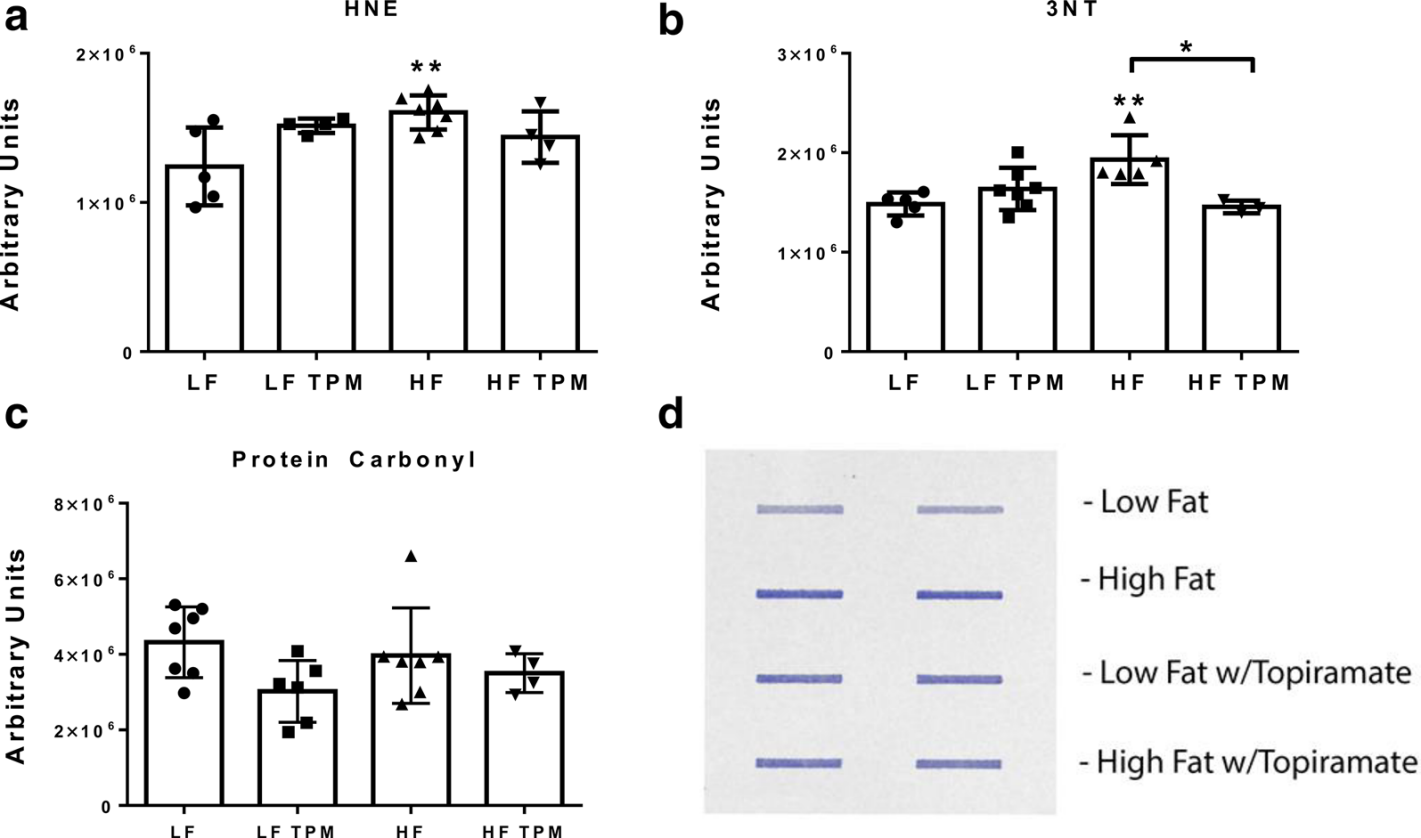
Effect of diet and topiramate on oxidative stress. Changes in 4- hydroxynonenal (HNE, **a**), 3-nitrotyrosine (3-NT, **b**), and protein carbonyls (**c**) levels in mouse brain homogenates from LF- and HF-fed CD-1 mice treated with or without TPM. HF diet led to a significant increase in HNE and 3-NT compared to LF controls. These increases were attenuated with TPM treatment. HF diet had no effect on protein carbonyls. The image in **d** is representative of the slot blot data used to collect these values. Values are expressed as arbitrary units, mean ± SD. Significance was determined by a one-way analysis of variance followed by Newman–Keuls post-test for HF vs. LF, LF vs. LF TPM, and HF vs. HF TPM. *p < 0.05; **p < 0.01

Oxidative stress was examined in hemibrain samples from CD-1 mice fed LF- and HF-diets and treated with TPM. Lipid peroxidation, one measure of oxidative stress, was assessed by measuring protein-bound 4-hydroxynonenal (HNE), a stable byproduct of lipid peroxidation. Elevated levels of protein-bound HNE are indicative of oxidative stress. Hyperglycemic HF-fed CD-1 mice had significantly increased (p < 0.01) levels of protein-bound HNE (Fig. 5a). Topiramate treatment partially prevented the increase in oxidative stress caused by HF diet consumption since HF-fed mice treated with TPM were not significantly different from the LF controls. Elevated levels of 3-NT, a marker of protein oxidation formed by peroxynitrite, which in turn is formed from reaction of nitric oxide with superoxide radical anions, is another measurement of oxidative stress. Hyperglycemic HF-fed CD-1 mice had significantly increased (p < 0.01) levels of 3-NT compared to LF controls (Fig. 5b). HF-fed TPM treated mice had significantly decreased (p < 0.05) levels of 3-NT compared to their HF-fed controls. There was no significant change in protein carbonyls, another measure of protein oxidation (Fig. 5c).

### Topiramate affects the tight junction proteins, claudin-12 and ZO-1

**Fig. 6:**
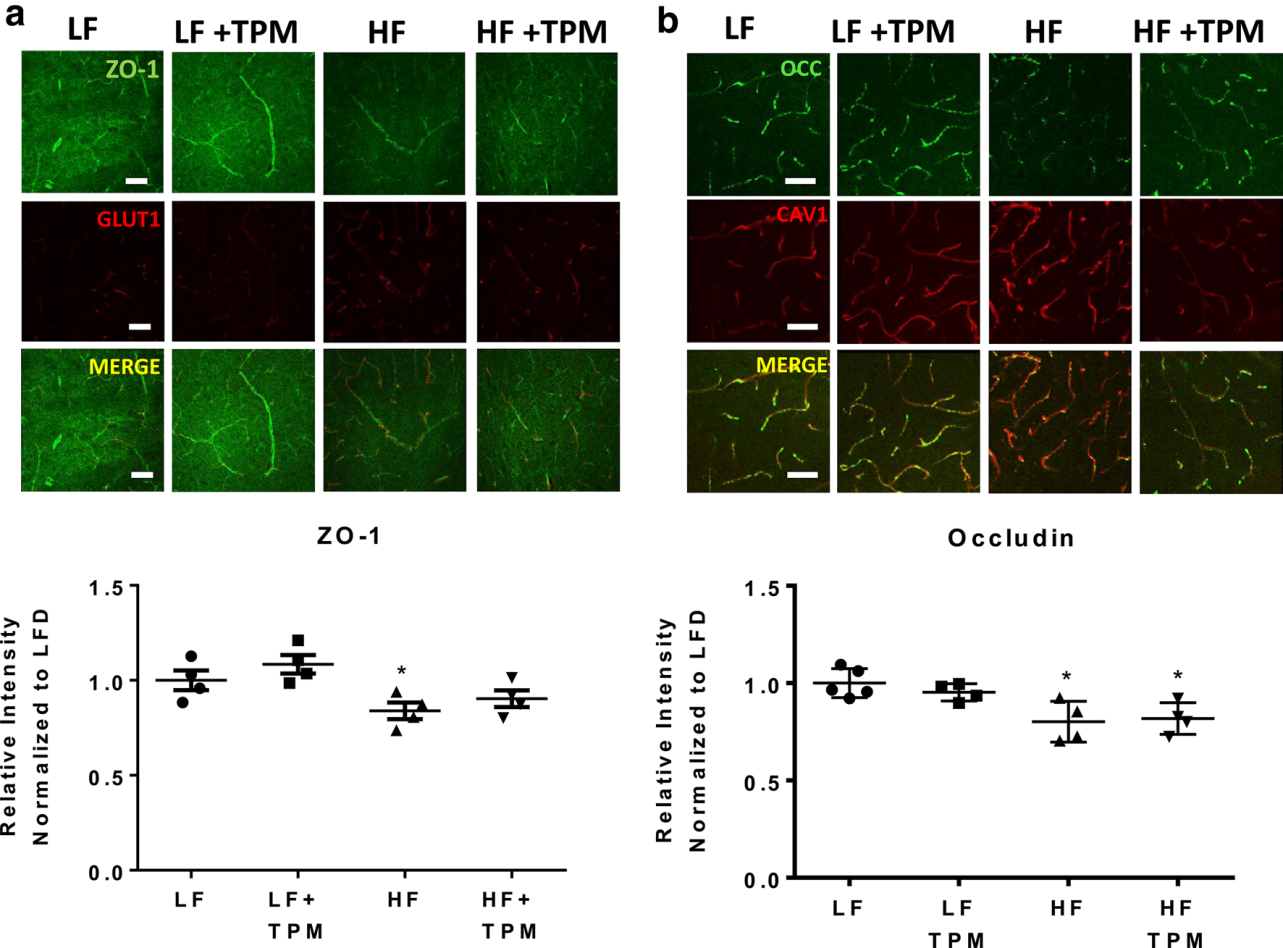
Effect of diet and topiramate on tight junction protein expression using immunofluorescence. Immunohistochemical techniques were used on tissue from the hypothalamus to examine ZO-1 (**a**) and occludin (**b**) expression in LF- and HF-fed CD-1 mice treated with or without TPM (**a**). ZO-1 and occludin expression were significantly decreased with HF-diet feeding compared to LF-fed controls. TPM treatment attenuated ZO-1 expression. GLUT1 was used as a vessel marker for ZO-1 and CAV1 as a vessel marker for occludin. The merged image localizes the tight junction staining to the vessels. Scale bar is 50 microns. Values are expressed as mean ± SD. Significance was determined by a one-way analysis of variance followed by Newman–Keuls post-test between HF vs. LF, LF vs. LF TPM, and HF vs. HF TPM. *p < 0.05

**Fig. 7:**
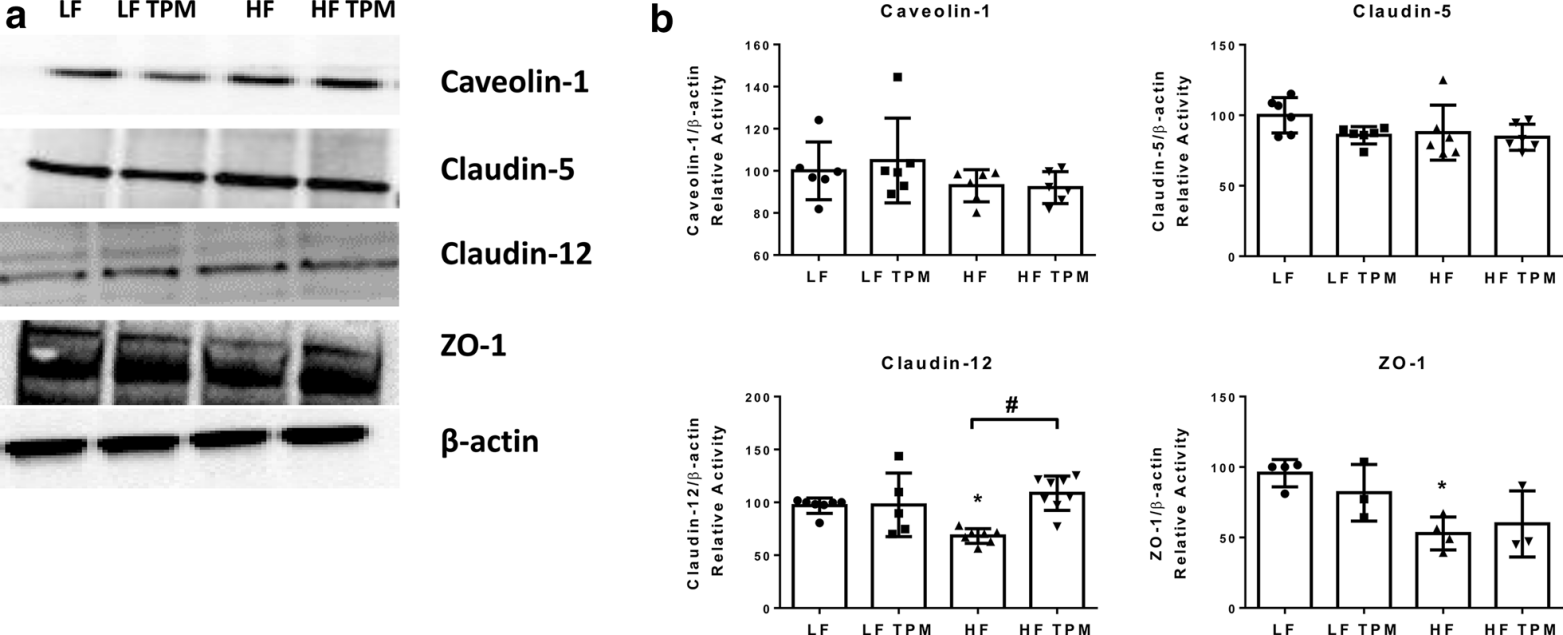
Effect of diet and topiramate on tight junction protein expression using immunoblot. Whole brain lysate was used to examine expression of the tight junction proteins, claudin-5, claudin-12, and ZO-1, and caveolin-1, the major structural protein of caveolae (**a**). HF-diet consumption led to a significant decrease in claudin-12 and ZO-1 expression which was attenuated with TPM treatment for claudin-12 (**b**). There were no significant changes in calveolin-1 or claudin-5. Values are expressed as mean ± SD and relative to the β-actin loading control. Significance was determined by a one-way analysis of variance followed by Newman–Keuls post-test between HF vs. LF, LF vs. LF TPM, and HF vs. HF TPM. *p < 0.05; ^#^p < 0.001

Immunohistochemical techniques were used to examine the tight junction proteins ZO-1 and occludin in the hypothalamus of CD-1 mice fed LF- or HF- diet treated with saline or TPM. HF-fed mice showed a significant decrease in ZO-1 (Fig. 6a) and occludin (Fig. 6b) fluorescent intensity in the hypothalamus (p < 0.05). Topiramate treatment partially prevented the decrease in ZO-1 expression caused by HF diet consumption since HF-fed mice treated with TPM were not significantly different from the LF controls. Whole brain lysate was used to measure protein expression of the tight junction proteins claudin-5, claudin-12, and ZO-1, relative to β-actin expression (Fig. 7). HF-diet fed mice showed a significant decrease in the tight junction proteins, claudin-12 (p < 0.05) and ZO-1 (p < 0.05, Fig. 7b). Topiramate treatment attenuated the effect of HF-diet feeding on the protein expression of claudin-12 (p < 0.05, Fig. 7b). HF-diet consumption had no significant effect on protein expression of claudin-5. In addition, we measured protein expression of caveolin-1, as it is the major structural protein of caveolae, the membrane microdomains involved in various aspects of vesicular trafficking [37, 38]. No change in caveolin-1 expression was observed.

## Discussion

Type II diabetes is associated with cognitive dysfunction resulting in microvascular and neurovascular unit changes. Type II diabetes is also associated with increased ROS and oxidative stress. Topiramate, a mCAi, has been shown to decrease ROS and oxidative stress in an STZ-induced mouse model of type I diabetes and to reverse cognitive impairment [15, 27, 28]. This approach has been effective in preventing BBB disruption in STZ-induced diabetes by preserving pericyte function [25, 27, 39, 40]. Here, we examined BBB and BRB disruption in a diet-induced obese model of type II diabetes. Two paradigms were used; in arm 1 (prevention), mice were treated with the mCAi TPM for 16 weeks concurrently with the onset of their LF or HF diets; in arm 2 (efficacy), the mice were fed their LF or HF diets for 20 weeks and then started on the 16 week TPM regimen while continuing their respective diets. As arm 1 examined the effects of TPM prior to the onset of BBB disruptions, this had a preventative aspect to it, whereas arm 2 examined effects of TPM after a time when BBB lesions are established and so had an efficacy aspect to it. Therefore, this experimental design was used to determine if TPM is effective both in the prevention of BBB disruption and as a potential treatment.

A potential confounder to this study would have been if TPM treatment had resulted in the reversal of diabetes. To examine this, we monitored the effect of TPM on body weight as it has been shown, when used in combination with other drugs, to reduce adiposity in humans and rodents and is currently being used as part of an anti-obesity medication regimen [41–43]. We demonstrated that TPM injections had no effect on body weight. This was similar to what we observed in studies of STZ-induced type I diabetes [25]. The mechanism by which TPM induces weight loss has yet to be elucidated. Potential mechanisms include decreasing food intake, reducing body fat gain, and increasing energy expenditure [42, 43]. We did not observe changes in body weight which could potentially be attributed to the relatively low dose of TPM (5 mg/kg vs. 10–100 mg/kg), the route of administration (subcutaneous injections vs. oral gavage), or that TPM was used as a single agent.

Topiramate has the potential to be an insulin sensitizing agent. This is apparent in rat models [44–46]. There is conflicting evidence of this in mouse models and human studies [25, 46–49]. Topiramate treatment had no effect on non-fasted blood glucose in our model, however, that does not imply it did not affect insulin sensitivity. Analysis of the diabetes panel to determine if the TPM treatment had an effect on metabolic hormones revealed that HF feeding caused an increase in GIP, leptin, and insulin levels and a decrease in ghrelin; consistent with the literature [50–53]. However, TPM treatment did not alter ghrelin, leptin, insulin, GIP, GLP-1, PAI-1, or resistin levels. Thus, the protective effects of TPM are not mediated by changes to the metabolic state of the animal. This is consistent with TPM protection being facilitated through its ability to inhibit mitochondrial carbonic anhydrase [15, 25].

Blood–brain barrier permeability was assessed using two radiolabeled ligands of varying sizes, ^99m^Tc-albumin (65 kDa) and ^14^C-sucrose (342 Da). The hypothalamus and hippocampus showed increased permeability to ^14^C-sucrose with HF-feeding. To date, no studies have examined permeability to a ligand this small in an in vivo type II diabetic model using radioactive techniques. Assessment was also completed using ^99m^Tc-albumin (65 kDa). We observed increases in ^99m^Tc-albumin in the whole brain after 16 weeks of HF-feeding. Increased permeability to ^99m^Tc-albumin was observed in the hypothalamus (at 16 weeks) and hippocampus (at 36 weeks). This was consistent with the literature which has used other large ligands, such as IgG (150 kDa) to examine BBB disruption [6–8]. Studies of obese animal models have shown disruption in the hypothalamus and hippocampus, similar to our findings in type II diabetes, which leads us to conclude that hormone levels may potentially drive BBB disruption more so than hyperglycemia. Transcytosis is a transcellular, vesicular based, molecular weight-independent form of capillary leakage, whereas paracellular transport is the intercellular, tight junction protein-dependent form of capillary leakage. There is no definitive method in which to distinguish the mechanism by which ^99m^Tc-albumin or ^14^C-sucrose crossed into each brain region, since both are capable of entering through paracellular and transcytotic routes. However, our studies have provided evidence that ^99m^Tc-albumin and ^14^C-sucrose cross into the brain of the diabetic mouse in distinct patterns from one another, implying that they are using different mechanisms of entry that are disrupted differentially.

Blood–retinal barrier disruption was also examined in this model. Diabetic retinopathy is a major complication associated with this disease and the major cause of blindness in Western countries. Here, we observed increased entry of ^99m^Tc-albumin into the retina simultaneously with increased entry into the whole brain at 16 weeks. This occurred prior to increased entry to the retina for ^14^C-sucrose which occurred at 36 weeks. That is increased permeability to ^14^C-sucrose in the retina was not found in arm one (16 weeks of HF diet), but was found in arm 2 (36 weeks of HF diet). Pericyte loss is a major hallmark of diabetic retinopathy, as well as in the brain microvasculature, and in other complications of type II diabetes [15, 54–57]. Topiramate treatment has proven efficacious in preventing pericyte loss in the brain of STZ-induced diabetic mice, but not in the retina [15, 25, 27]. This suggests that the mechanism of disruption in the BRB is distinct to that of the BBB. Also, that the mechanism of pericyte loss in the two tissue types may be independent of one another. The actions of the mCAi in lowering ROS may not be the appropriate target to preventing pericyte loss in the retina.

High-fat diet consumption is associated with increased oxidative stress. Oxidative stress affects BBB integrity [17–19]. Studies have shown that brain levels of HNE and 3-NT, markers of lipid peroxidation and protein oxidation, respectively, are increased in brain tissue, and other tissues, including adipose and liver after HF-diet consumption [58–63]. Protein carbonyls have been shown to be increased in the brain with specific types of HF-diets which include lard [64] and TPM treatment has been shown to reduce these markers of oxidative stress in STZ-induced diabetic mice [15, 28]. Topiramate treatment of the hyperglycemic type II diabetic mice in this study demonstrated that it was efficacious in reducing oxidative stress in this model. Decreasing oxidative stress is a mechanism by which TPM acts to improve BBB integrity and TPM has been shown to act on pericytes to decrease reactive oxygen species and improve cellular respiration in the presence of high glucose [39, 40].

In regions of the brain where BBB integrity has been disrupted, such as the hippocampus, studies have shown that tight junction protein expression is decreased [29, 30]. However, when regions not affected by HF diet consumption are examined, such as the parietal cortex, or at the whole brain level, the effect on tight junction expression is often absent [6, 31]. In this study, we had access to hemibrains of mice shown to have BBB disruption, for the preparation of lysate to examine the effect of HF-diet consumption and TPM treatment on tight junction protein expression. We demonstrated that HF-diet led to a decreased expression of ZO-1, claudin-12 and occludin, which was attenuated or partially attenuated with TPM treatment. Examining tight junction protein expression in specific regions where BBB disruption has occurred may implicate other tight junction proteins in this model. This provides a second mechanism by which HF-diet consumption leads to a decline in BBB integrity and TPM is able to attenuate the effect.

Obesity has been associated with disorders affecting the CNS including depression and cognitive impairment. Cognitive dysfunction has been linked to the consumption of a Western diet, a diet that contains both high levels of fat (35–60% total kcal) and added sugars. While much of the research done on obesity has focused on its effect in the hypothalamus, it is now understood that obesity also affects other brain regions including the hippocampus [65]. The hippocampus, a brain region associated with the control of certain learning and memory processes, is particularly vulnerable to Western diet consumption. This study has demonstrated that the disruption of the hypothalamus and hippocampus are evident in animal models of type II diabetes as well as with obesity. The findings of this study demonstrate that the pattern of BBB disruption in type II diabetes is different to that observed in type I diabetes [25]. Type I diabetic animal models showed disruption to ^14^C-sucrose (342 Da) in the cortices, thalamus, and midbrain [25, 26], while in our diet-induced obese type II diabetic model we showed disruption in the hypothalamus and hippocampus. This supports findings that cognitive impairment between type I and type II diabetics differ from one another [66, 67]. Cognitive dysfunction in type I diabetes is characterized by a slowing of mental speed and diminished mental flexibility, while learning and memory are spared, while in type II diabetes, learning and memory are the primary issues of cognitive impairment. Unfortunately, in a recent study, Espeland et al. demonstrated that long-term intensive lifestyle intervention, using diet and exercise, in type II diabetics had no effect on cognitive function, despite improvements in health [68]. This supports our belief that hormone levels play a stronger role in determining BBB disruption and subsequent cognitive impairment.

## Conclusion

Blood–brain barrier and blood–retinal barrier dysfunction were examined in a mouse model of diet-induced obese type II diabetes. Mitochondrial carbonic anhydrase inhibition, using the drug topiramate, was used as potential prevention and treatment strategy in this model. Increased permeability to ^14^C-sucrose was detected in the hypothalamus and hippocampus, and increased permeability to ^99m^Tc-albumin in the whole brain. In the retina, increased entry of ^99m^Tc-albumin was detected at 16 weeks while increased entry to ^14^C-sucrose was not detected until 36 weeks. Our studies indicate that topiramate is efficacious at both preventing and treating BBB disruption in the brain, but not retina, in this diet-induced obese type II diabetic mouse model. These studies demonstrate that there are spatial and temporal differences in ^14^C-sucrose and ^99m^Tc-albumin permeability in the brain and retina of diet-induced obese type II diabetic mice.

## Abbreviations

3-NT: 3-nitrotyrosine
BBB: blood–brain barrier
BRB: blood–retinal barrier
CNS: central nervous system
HNE: 4-hydroxynonenal
mCA: mitochondrial carbonic anhydrase
ROS: reactive oxygen species
STZ: streptozotocin
TPM: topiramate
